# Cloacal Exstrophy Associated with a Hindgut Duplication Anomaly: A Case Report of Challenges in Hindgut Preservation

**DOI:** 10.1055/a-2544-3341

**Published:** 2025-03-18

**Authors:** Suliaman Alaqeel, Jamila Almaary, Fatmah Alrabodh, Fayez AlModhen

**Affiliations:** 1 Department of Pediatric Surgery, Ministry of National Guard Health Affairs, Riyadh, Riyadh, Saudi Arabia; 2 Department of Pediatric Urology, Ministry of National Guard Health Affairs, Riyadh, Riyadh, Saudi Arabia

**Keywords:** hindgut, intestinal duplication, cloacal exstrophy

## Abstract

Cloacal exstrophy (CE) is a rare condition, and the preservation of a short hindgut is crucial for growth, fluid-electrolyte balance, and bowel management. Herein, we present the case of an infant with concurrent anomalies and the challenges faced during the preservation of both hindguts. A preterm male infant, born at 34 weeks weighing 2 kg, was diagnosed with CE. The first stage of CE repair was performed at 3 months of age, involving the separation and tubularization of the cecal plate and the joining of the two hemi-bladders. During the procedure, hindgut duplication was noted. Each hindgut had a short mesentery with far-separated, tiny blind ends (7 and 10 cm in length), each directed toward opposite sides of the pelvis and measuring 8 Fr in caliber. Due to the risk of compromising the blood supply during rotation and mobilization of one hindgut, the decision was made to exteriorize each hindgut as end stomas. Both stomas began functioning as expected. However, a colo-cutaneous fistula (connecting the cecum to the midline surgical wound) developed and was managed conservatively for 8 weeks. During this period, despite the functioning left stoma, the right stoma was stenosed, and the fistula persisted. The infant underwent a second laparotomy for fistula repair and reconstruction of both hindguts. This was successfully managed by creating a single-lumen end stoma, preserving the entire length of the hindguts with a wider caliber. In conclusion, complex cases of CE are uncommon, and unexpected operative findings should always be anticipated. Reconstructing both hindguts into a single lumen during the initial procedure can be challenging but is achievable with time and careful effort. Preservation of both hindgut ends should be prioritized. However, long-term outcomes remain uncertain due to the rarity of this presentation.

## Introduction

Cloacal exstrophy (CE) is a rare complex anomaly with an incidence rate of 1:300,000. 1 2 Historically, management goals have focused on survival due to high mortality secondary to sepsis, electrolyte imbalance, and malnutrition. 1 3 With current advances in care and multidisciplinary approaches, the survival rate is 83 to 100%. 1 3 Consequently, the emphasis of care has evolved toward enhancing the quality of life and optimizing the functional outcomes for patients. 4 Shifting the surgical management of gastrointestinal initial reconstruction from intestinal diversion (ileostomy) to hindgut preservation with end colostomy placement has dramatically improved survival, bowel growth, and adaptation; moreover, future options include pull-through procedures and genitourinary reconstruction utilizing the intestine. 5

CE is associated with gastrointestinal, genitourinary, spine, and limb anomalies. 3 Common associations include short hindgut, appendix duplication, bladder duplication, and uterine duplication. 3 Variable hindgut lengths ranging from 2 to 20 cm and slightly longer have been reported and described. 4 6 In the general population, colon and rectal duplications represent 16% and 4% of total enteric duplications, respectively. 7 To the best of our knowledge, hindgut duplication in CE patients has been described previously in only two patients. Herein, we present an infant with similar concurrent anomalies and the challenges faced in preserving both hindguts.

## Case Presentation

**Fig. 1:**
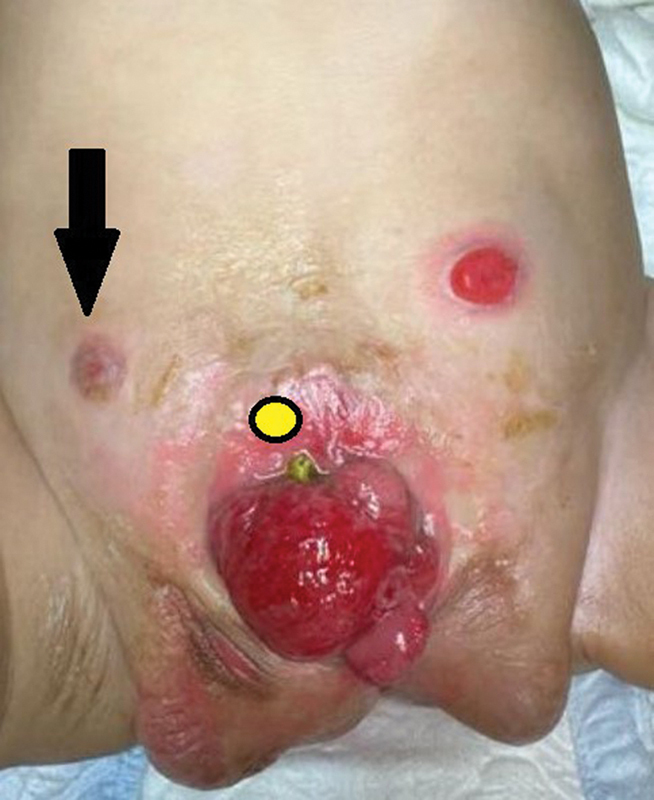
Black arrow: stenosed right stoma. Yellow circle: colo-cutaneous fistula site.

**Fig. 2:**
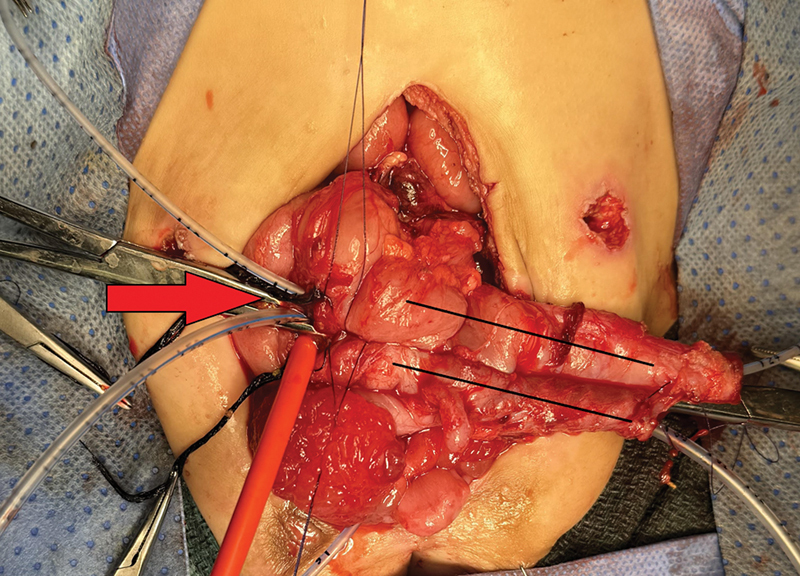
Two black lines: hindguts adjoined together with side-to-side anastomosis. Red arrow: site of the colo-cutaneous repair.

In a preterm 34-week-old, 2 kg, male (chromosomal analysis: XY) born with CE and the OEIS complex consisting of omphalocele, bladder exstrophy, imperforate anus, closed spinal dysraphism with lumbosacral lipomyelomeningiocele and low-lying tethered cord. First-stage CE repair was performed at 3 months of life, with separation and tubulization of the cecal plate, followed by adjoining the two hemi-bladders. Hindgut duplication was noted intraoperatively, and each blind end measured 7 and 10 cm long, with a width of 8 Fr caliber feeding tube. Each hindgut had a separate short mesentery directed separately to the pelvis sides, making a 180-degree angle between them. A trial of rotating and mobilizing one hindgut (right) was not possible, as it would jeopardize its blood supply. Thus, a decision was made to exteriorize each hindgut separately as a stoma. Both stomas started functioning as anticipated; however, a colo-cutaneous fistula developed (cecum to midline surgical wound) and was managed conservatively for 8 weeks. During this waiting time, despite the function of the left stoma, the right stoma stenosed, and the colo-cutaneous fistula persisted ( Fig. 1 ). The patient underwent a second laparotomy for fistula repair as well as reconstruction of the hindgut. During the second laparotomy, the hindgut was wider in caliber and easier to manipulate compared with the first procedure. Both hindguts were adjoined together, and side-to-side anastomosis was performed ( Fig. 2 ). A single left-end colostomy was then created. The patient was scheduled for long-term follow-up with a multidisciplinary team, including pediatric surgery, urology, neurosurgery, general pediatrics, and a clinical dietitian.

## Discussion

The quality of life, growth, and hospitalization of patients with CE, especially in the first year of life, are affected by the type of gastrointestinal reconstruction, which includes ileostomy or hindgut preservation, cecal tabularization, and colostomy. 6 8 9 Saving the hindgut and avoiding ileostomy has prevented medical morbidities and mortalities secondary to metabolic derangement, dehydration, frequent hospitalizations, TPN dependence, and sepsis. 9 The surgical principles of preserving and preserving the hindgut regardless of its length and maintaining its continuity with the gastrointestinal system through cecal tuberization are well emphasized. 5 However, when facing duplication of the hindgut, new technical challenges can be encountered.

Rickham reported the first patient with hindgut duplication; both were removed, and the ileum was pulled through the perineum. 10 The patient died in 2 months of dehydration and failure to thrive. A better option is to preserve both hindguts and unify them together with side-to-side anastomosis and create a single-end stoma, as reported by Tirell et al. 7 In the report, the patient had duplicated hindguts, each with its own mesenteric blood supply. Specifically, one is directed toward the pelvis at the midline, and the other is directed toward the left paracolic gutter. Thus, 90-degree angle separation was performed, which was technically feasible at the initial surgery, to join them, and the patient survived. However, in our case, technically, both hindguts were in opposite directions, resulting in a 180-degree angle between them. An attempt to mobilize one side to join the other side was not possible, as it compromised the blood supply. Both hindguts were preserved, and two end stomas were created. Stenosis of the right end stoma secondary to decreased blood supply was a consequence of that attempt at the initial surgery.

When faced with duplicated hindguts in CE patients, one option is to resect one of the hindguts while preserving the other. If resection is deemed easier and safer, it can still be challenging to decide which hindgut to keep and which hindgut to remove, especially given uncertainties about which hindgut will function properly. Preserving both hindguts allows the fecal stream to flow through their lumens, giving each hindgut an equal opportunity for growth. Interestingly, this was observed during the second surgery in our patient. Sixty days after the initial procedure, we successfully performed an anastomosis of the two hindguts, facilitated by their increased caliber and extended mesentery. Afterward, the patient did very well, gained weight, and underwent bladder closure with bilateral osteotomy at the age of 12 months. The risk of malignancy in cases of gut duplication has been primarily documented in instances where the duplicated segment is isolated from the fecal stream and lacks continuity with the rest of the bowel. This isolation can lead to chronic inflammation and an increased potential for neoplastic changes, which was not the case in our reported case. 7

In conclusion, complex CE cases are rare, and unusual operative findings should always be anticipated. Reconstructing both hindgut ends to one end in the first setting can be challenging but possible with time, and an attempt to preserve both ends should be made. Long-term outcomes cannot be determined due to the rarity of such a presentation.

## Notes

Importance for a Pediatric Surgeon Üloacal exstrophy is extremely rare, and surgeons should expect unusual findings during surgery. Reconstructing both hindgut ends into one is challenging but possible, and preserving both ends is preferred. Long-term outcomes are hard to predict due to the condition's rarity.
